# Finger tapping impairments are highly sensitive for evaluating upper motor neuron lesions

**DOI:** 10.1186/s12883-017-0829-y

**Published:** 2017-03-21

**Authors:** Afsaneh Shirani, Braeden D. Newton, Darin T. Okuda

**Affiliations:** Department of Neurology and Neurotherapeutics, Clinical Center for Multiple Sclerosis, Multiple Sclerosis & Neuroimmunology Imaging Program, Neuroinnovation Program, University of Texas Southwestern Medical Center, Dallas, TX 75390 USA

**Keywords:** Finger tapping, Neurological examination, Upper motor neuron, Multiple sclerosis, Demyelinating, Magnetic resonance imaging

## Abstract

**Background:**

Identifying highly sensitive and reliable neurological exam components are crucial in recognizing clinical deficiencies. This study aimed to investigate finger tapping performance differences between patients with CNS demyelinating lesions and healthy control subjects.

**Methods:**

Twenty-three patients with multiple sclerosis or clinically isolated syndrome with infratentorial and/or cervical cord lesions on MRI, and 12 healthy controls were videotaped while tapping the tip of the index finger against the tip and distal crease of the thumb using both the dominant and non-dominant hand. Videos were assessed independently by 10 evaluators (three MS neurologists, four neurology residents, three advanced practice providers). Sensitivity and inter-evaluator reliability of finger tapping interpretations were calculated.

**Results:**

A total of 1400 evaluations (four videos per each of the 35 subjects evaluated by 10 independent providers) were obtained. Impairments in finger tapping against the distal thumb crease of the non-dominant hand, identified by neurologists, had the greatest sensitivity (84%, p < 0.001) for detecting impairment. Finger tapping against the thumb crease was more sensitive than the thumb tip across all categories of providers. The best inter-evaluator reliability was associated with neurologists’ evaluations for the thumb crease of the non-dominant hand (kappa = 0.83, *p <* 0.001).

**Conclusions:**

Impaired finger tapping against the distal thumb crease of the non-dominant hand was a more sensitive technique for detecting impairments related to CNS demyelinating lesions. Our findings highlight the importance of precise examinations of the non-dominant side where impaired fine motor control secondary to an upper motor injury might be detectable earlier than the dominant side.

## Background

The neurological examination remains the cornerstone of neuroanatomic localization and diagnosis of nervous system disorders, despite breakthrough advances in neuroimaging [1]. However, an unmet need exists for high quality research on neurological examination techniques aimed at identifying the most pragmatic, time-effective, and reliable components with a high degree of sensitivity for appreciating central nervous system (CNS) abnormalities that may be utilized by a wide variety of healthcare providers [2].

Repetitive rapid finger tapping is a common test of fine motor control of the upper extremities. Normal finger tapping requires the functional integrity of the corticospinal tract, cerebellar motor circuitry, and proprioceptive pathways [3]. Tasks involving the tapping of fingers, with varying techniques, have been widely studied in various domains such as neuropsychiatry and behavioral neurology (as a predictor of IQ and reaction time) [4, 5], traumatic brain injury and stroke (as an indicator of motor recovery) [6, 7], and perhaps most commonly in Parkinsonism (as an index of bradykinesia and hypokinesia) [8]. Upper extremity dysfunction has been reported in up to 80% of patients with multiple sclerosis (MS), the most common demyelinating disease of the central nervous system (CNS) [9]. Impairments of manual fine motor skills can significantly impact the quality of life of patients with MS. A number of studies have specifically examined upper extremity-related fine motor control tasks in patients with MS [10–12]. Evaluating upper extremity dysfunction is more difficult compared to the lower limbs given the higher complexity and multidimensionality of manual tasks [10]. The complex neural substrate for motor control in demyelinating diseases remains to be fully understood. Recent neuroimaging evidence from patients with long-standing MS suggests that the mean upper cervical cord area, and the presence of cervical and infratentorial lesions, appear to be predictors of motor dysfunction, having the potential to be easily used in the real-world clinic practice as predictive metrics for physical function [13].

Here, we studied the validity of the repetitive finger tapping test (in both the dominant and non-dominant hand) in subjects with and without inflammatory demyelinating lesions within the infratentorial region and cervical spinal cord. Three categories of providers (i.e. MS neurologists, neurology residents, and advanced practice providers (APPs)) with different levels of training and skills were included to account for inter-evaluator variability with respect to the interpretation of the finger tapping tests. We also assessed for performance impairments in tapping of the index finger against the thumb tip in comparison to the distal interphalangeal joint of the thumb (distal thumb crease), two different techniques commonly used within the clinical setting. We hypothesized that finger tapping is a sensitive test to identify CNS demyelinating lesions and its sensitivity could be affected by the technique used as well as the provider performing the test.

## Methods

### Design and study population

**Fig. 1:**
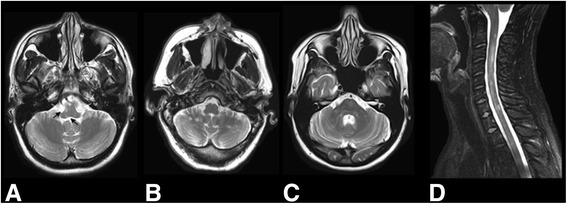
Axial T2-weighted MRI images of the infratentorial region from three unique patients demonstrating foci of T2-hyperintensity at the brainstem (right pontomedullary sulcus and medial vestibular nucleus (arrows)) (**a**), bilateral cerebellar lobes (**b**), and brainstem and bilateral cerebellar peduncle regions (**c**). Sagittal T2-weighted MRI image of the cervical spinal cord revealing multi-focal regions of T2-hyperintensity within the parenchyma (**d**)

This study included 23 adult patients (age > 18 years) with MS or clinically isolated syndrome (CIS) [14], and 12 healthy adult control subjects. Patients were consecutively recruited from the UT Southwestern Clinical Center for Multiple Sclerosis between June 2014 and October 2014. Selection criteria for patients required the presence of ≥ 1 infratentorial (brainstem, cerebellar peduncle, or cerebellar lesions) or cervical spinal cord lesions on MRI based on the formal interpretation of brain and cervical spinal cord MRI by a neuroradiologist with subsequent verification by an MS neurologist (see Figure 1). Healthy controls had no known history of neurological disorders, and were recruited from clinic staff or family members and friends of patients enrolled over the same time period.

### Data collection

Each of the 35 study subjects were separately videotaped and asked to perform the finger tapping test by tapping the i) index finger on the thumb tip using the dominant hand, ii) index finger on the thumb tip using the non-dominant hand, iii) index finger on the distal thumb crease using the dominant hand, and iv) index finger on the distal thumb crease using the non-dominant hand. Participants were provided with instructions to repetitively tap the index finger on the thumb for at least 20 s, an adopted and modified technique from the Unified Parkinson’s Disease Rating Scale (UPDRS) instructions which require tapping as quickly and as big as possible [8]. Each hand was tested separately. A total of 140 videos (four videos per each of the 35 subjects) of finger tapping performance were subsequently collected. All videos were edited to capture only the first 10 s of finger tapping performance in order to better simulate how finger tapping is evaluated in the real-world clinical practice. The sequence of videos was randomized and a standardized test module was created for evaluators.

The videos were assessed independently by 10 evaluators of different levels of experience, and skills in neurology (three MS neurologist, four neurology residents, and three APPs) who were all blinded to the study subjects. The evaluators were first trained on how to score finger tapping using the UPDRS [8], the most commonly used rating scale for finger tapping performance, by viewing sample videos. Since younger age, male sex, and the dominant hand have been suggested by some studies to be associated with higher tapping rates [15], information on patient’s sex, age and hand dominance were provided to the evaluators before assessing each video. Repeated viewing of the videos was allowed if the evaluator was not able to rate the finger tapping on the first attempt.

### Outcomes, and comparisons

The main outcome included the identification of impairments in finger tapping performance related to the presence of inflammatory demyelinating infratentorial and cervical spinal cord lesions. Separate comparisons with sensitivity determinations were made when finger tapping was performed against the tip in comparison to the distal crease of the thumb, with the dominant and non-dominant hand, and also across three categories of providers (MS neurologists, neurology residents, and APPs). Other measures of validity including specificity and accuracy were also analyzed. Results were also compared when findings for the dominant and non-dominant hands were combined (i.e. when finger tapping was considered abnormal if either or both of the dominant and non-dominant hands were evaluated as abnormal). In addition, we assessed the inter-evaluator reliability of finger tapping test within each category of the providers. We also looked into the sensitivity of other methods of assessing motor function including manual muscle testing (graded 0 to 5) performed by an MS specialist [3].

### Statistics

Baseline characteristics of patients and healthy control subjects were compared using Fisher’s exact test for categorical variables and *t*-test for quantitative variables. For the purpose of calculating measures of validity, all finger tapping interpretations were classified in a binary manner as normal (score 0 on UPDRS) vs. abnormal (score ≥1). Chi-square or Fisher’s exact tests (where appropriate), and the associated probability values, were used to calculate sensitivities and specificities. Inter-evaluator reliability of finger tapping interpretation among the providers within the same category was calculated by determining the level of agreement between interpretations using Cohen’s kappa coefficient. Kappa statistics were graded as follows based on Fleiss’s scale [16]: values <0.40 poor agreement; 0.40-0.75 fair to good agreement; ≥0.75 excellent agreement.

All statistical tests were two-sided, and *p* < 0.05 was considered statistically significant. Statistical analyses were performed using the Statistical Package for the Social Sciences (SPSS Inc. Chicago, Illinois, version 16.0).

## Results

A total of 35 subjects (23 patients with MS or CIS, and 12 healthy control subjects) were included. Table 1 shows the baseline characteristics of the patients and healthy controls. Of the 23 patients, 19 had MS, and four had CIS at the time of the study. The majority of the patients (65%) had both infratentorial and cervical spinal cord lesions. There was no significant difference in handedness between the patients and controls.

**Table 1:** Baseline characteristics of patients with infratentorial or cervical spinal cord demyelinating lesions in comparison to healthy control subjects

Baseline characteristics	Patients (*N =* 23)	Healthy controls (*N =* 12)	*P* value
Sex, N(%)			
Male	6 (26%)	8 (67%)	0.31^a^
Female	17 (74%)	4 (33%)	
Age (years)			
Mean (SD)	44.8 (14.6)	37.4 (14.5)	0.87^b^
Range	22 – 74	22 – 62	
Ethnicity, N(%)			
White	22 (96%)	11 (92%)	0.57^a^
African American	1 (4%)	0 (0%)	
Middle Eastern	0 (0%)	1 (8%)	
Handedness, N(%)			
Right	21 (91%)	11 (92%)	0.73^a^
Left	2 (9%)	1 (8%)	
Disease subtype, N(%)			
CIS	4		
RRMS	17		
SPMS	1		
PPMS	1	-	-
Age at onset of disease symptoms (years)			
Mean (SD)	36.5 (14.7)	-	-
Range	16 – 68		
Disease duration from onset of symptoms (years)			
Mean (SD)	8.7 (9.1)	-	-
Range	0 – 39		
Lesion location on MRI, N^c^			
Infra-tentorial	20	-	-
Brainstem	15		
Cerebellum	18		
Cervical spinal cord	18		

^a^Two-sided Fisher’s exact test

^b^Two sided *t*-test

^c^Data reflect the presence of multi-focal lesions within the CNS

A total of 1400 evaluations (4 videos corresponding to four settings of the test per each of the 35 subjects viewed by 10 evaluators) were analyzed including 420 evaluations by neurologists, 560 evaluations by neurology residents, and 420 evaluations by APPs. Table 2 shows the sensitivity, specificity, and accuracy of the finger tapping test in identifying infratentorial and cervical cord demyelinating lesions in four different settings of finger tapping depending on whether the test was performed using the dominant vs. non-dominant hand, and against thumb tip versus the distal thumb crease across different categories of providers. Finger tapping against the thumb tip using the dominant hand was found to have an overall sensitivity of 69.6% (*p =* 0.003), 45.7% (*p =* 0.859), and 42% (*p =* 0.004) among neurologist, neurology residents, and APPs, respectively. When tapping was performed over the distal thumb crease of the dominant hand, the corresponding sensitivity values increased to 78.3% (*p <* 0.001), 71.7% (*p =* 0.026), and 62.3% (p < 0.001) for neurologists, neurology residents, and APPs, respectively. When results for non-dominant hands were analyzed, finger tapping using the thumb tip of the non-dominant hand was found to have a sensitivity of 78.3% (p < 0.001), 56.5% (*p =* 0.021), and 43.5% (*p <* 0.001) among neurologists, neurology residents, and APPs. When tapping was performed over the distal thumb crease of the non-dominant hand, the corresponding sensitivity values increased to 84.1% (*p <* 0.001), 70.7% (*p =* 0.006), and 72.5% (*p <* 0.001) for neurologist, neurology residents, and APPs. Overall when evaluations by all categories of providers were combined, finger tapping using the distal thumb crease of the non-dominant hand was still found to have the highest sensitivity value compared to other settings (Table 2). In addition, interpretations of finger tapping test made by neurologists were found to be associated with higher sensitivities compared to neurology residents and APPs (Table 2).

**Table 2:** Validity of the finger tapping test for identifying infratentorial and cervical cord demyelinating lesions in four different settings based on handedness, and location of tap (i.e. thumb tip versus distal thumb crease) across different categories of providers (neurologists, neurology residents, and advanced practice providers)

	Dominant hand, Thumb tip				Dominant hand, Distal thumb crease				Non-dominant hand, Thumb tip				Non-dominant hand, Distal thumb crease			
	Sensitivity	Specificity	Accuracy	*P* value^a^	Sensitivity	Specificity	Accuracy	*P* value^a^	Sensitivity	Specificity	Accuracy	*P* value^a^	Sensitivity	Specificity	Accuracy	*P* value^a^
Neurologist 1	69.6%	66.7%	68.6%	0.040^b^	87.0%	58.3%	77.1%	0.015	78.3%	75.0%	77.1%	0.004	82.6%	75.0%	80.0%	0.002
Neurologist 2	60.9%	83.3%	68.6%	0.013^b^	82.6%	83.3%	82.9%	<0.001	78.3%	100.0%	85.7%	<0.001^b^	82.6%	66.7%	77.1%	0.007
Neurologist 3	52.2%	91.7%	65.7%	0.013	87.0%	58.3%	77.1%	0.015	73.9%	100.0%	82.9%	<0.001^b^	91.3%	75.0%	85.7%	<0.001
All neurologists	69.6%	61.1%	66.7%	0.003^b^	78.3%	66.7%	74.3%	<0.001^b^	78.3%	86.1%	81.0%	<0.001^b^	84.1%	83.3%	83.8%	<0.001^b^
Neurology resident 1	43.5%	75.0%	54.3%	0.463	73.9%	83.3%	77.1%	0.001^b^	56.5%	75.0%	62.9%	0.076^b^	82.6%	66.7%	77.1%	0.007
Neurology resident 2	47.8%	41.7%	45.7%	0.555^b^	69.6%	41.7%	60.0%	0.709	47.8%	58.3%	51.4%	0.728^b^	65.2%	50.0%	60.0%	0.477
Neurology resident 3	43.5%	33.3%	40.0%	0.193^b^	73.9%	41.7%	62.9%	0.451	56.5%	58.3%	57.1%	0.404^b^	65.2%	33.3%	54.3%	1.000
Neurology resident 4	47.8%	58.3%	51.4%	0.728^b^	69.6%	25.0%	54.3%	1.000	65.2%	66.7%	65.7%	0.072^b^	69.6%	66.7%	68.6%	0.040^b^
All neurology residents	45.7%	52.1%	47.9%	0.859^b^	71.7%	47.9%	63.6%	0.026^b^	56.5%	64.6%	59.3%	0.021^b^	70.7%	54.2%	65.0%	0.006^b^
APP 1	52.2%	91.7%	65.7%	0.013	56.5%	100%	71.4%	0.001	39.1%	100%	60.0%	0.015	73.9%	91.7%	80.0%	<0.001^b^
APP 2	34.8%	91.7%	54.3%	0.121	69.6%	100%	80.0%	<0.001^b^	47.8%	91.7%	62.9%	0.027	82.6%	66.7%	77.1%	0.007
APP 3	39.1%	75.0%	51.4%	0.476	60.9%	91.7%	71.4%	0.003^b^	43.5%	83.3%	57.1%	0.149	60.9%	100.0%	74.3%	0.001
All APPs	42.0%	86.1%	57.1%	0.004^b^	62.3%	97.2%	74.3%	<0.001^b^	43.5%	91.7%	60.0%	<0.001^b^	72.5%	86.1%	77.1%	<0.001^b^
Overall	51.7%	65.0%	56.3%	0.003^b^	70.9%	68.3%	70.0%	<0.001^b^	59.1%	79.2%	66.0%	<0.001^b^	75.2%	72.5%	74.3%	<0.001^b^

*APP* Advanced practice provider

^a^Two-sided Fisher’s exact test unless indicated otherwise

^b^Two-sided chi-square test

Table 3 displays the inter-evaluator reliability of the finger tapping test within each of the three categories of providers across different techniques. Overall, the highest kappa values were associated with evaluations by neurologists (0.83 with *p <* 0.001 for the distal thumb crease of the non-dominant hand, and 0.82 with *p <* 0.001 for the distal thumb crease of the dominant hand), indicating an excellent inter-evaluator agreement. Among neurologists and APPs, the kappa values associated with the distal thumb crease were consistently higher than the ones associated with the thumb tip for both the dominant and non-dominant hands. However, among neurology residents, this effect was only found for the non-dominant hand.

**Table 3:** Inter-evaluator reliability of the finger tapping test in four different settings based on handedness and location of tap (thumb tip versus distal thumb crease) within different categories of providers (neurologists, neurology residents, and advanced practice providers)

	Dominant hand, Thumb tip		Dominant hand, Distal thumb crease		Non-dominant hand, Thumb tip		Non-dominant hand, Distal thumb crease	
	Kappa	*P* value	Kappa	*P* value	Kappa	*P* value	Kappa	*P* value
Neurologists (*N =* 3)	0.73	<0.001	0.82	<0.001	0.69	<0.001	0.83	<0.001
Neurology residents (*N =* 4)	0.53	<0.001	0.38	<0.001	0.48	<0.001	0.64	<0.001
APPs (*N =* 3)	0.56	<0.001	0.72	<0.001	0.38	0.002	0.54	<0.001
Overall (*N =* 10)	0.47	<0.001	0.43	<0.001	0.38	<0.001	0.56	<0.001

*APP* advanced practice provider

The specificity of finger tapping using the thumb tip of the dominant hand increased from 61.1% (*p =* 0.003) to 66.7% (p < 0.001) for the distal thumb crease of the dominant hand among neurologists, and from 86.1% (*p =* 0.004) to 97.2% (*p <*0.001) among APPs; however, this effect was not consistently seen in other settings of the tests (Table 2). With respect to other methods of assessing motor function, impaired manual muscle testing (graded 0 to 5) was found to have a sensitivity of 45.5% (*p =* 0.053) for identifying upper motor neuron lesions.

## Discussion

Our findings suggest that the specific technique of finger tapping against the distal thumb crease was more sensitive for identifying upper motor neuron injury due to demyelinating lesions when compared to tapping against the thumb tip. We also identified that finger tapping using the non-dominant hand was associated with greater sensitivities amongst all healthcare providers in identifying a CNS demyelinating focus as compared to when the technique was executed with the dominant hand. Finger tapping against the distal thumb crease of the non-dominant hand, when evaluated by neurologists, was associated with the highest sensitivity (84%, *p <* 0.001), and inter-evaluator reliability (kappa = 0.83, *p <* 0.001), when compared to all the other techniques of performing the test, and other categories of providers (neurology residents and APPs).

Repetitive rapid finger tapping is viewed classically as an assessment of fine motor skills of the upper extremities that requires coordinated alternating activity of distal flexor and extensor muscles. Fine independent finger movements are crucial for many motor skills of everyday life. Quantitative assessment of a repetitive finger to-thumb tip-opposition task was recently found to be highly valuable in discriminating MS patients with very low Expanded Disability Status Scale (EDSS) from healthy controls [12]. While finger tapping is traditionally examined in clinical practice by repetitive tapping of the index finger against the thumb tip, our study represents a first effort to look into an infrequently utilized variation involving tapping against the distal thumb crease. The observation of a higher sensitivity of finger tapping against the distal thumb crease compared to the thumb tip for detection of CNS demyelinating lesions could possibly suggest greater recruitment of finger and wrist extensor muscles when tapping is performed against the distal thumb crease. Kinematics and muscle activation pattern of finger tapping have been the subject of several previous studies [17, 18]. However, these prior studies simplified the task by tapping with the index finger on a computer keyswitch [17], making the extrapolation of findings to our study challenging. Our findings do provide evidence to suggest the need for refinement of existing traditional assessments as well as the clinical training delivered to healthcare providers.

The finding that tapping with the non-dominant hand was associated with an overall higher sensitivity for identifying CNS demyelinating lesions compared to the dominant hand (regardless of whether tapping was performed against the thumb tip or the distal thumb crease), may suggest a lower “reserve capacity” in the motor networks responsible for controlling the non-dominant side. Historically, within the cognitive sciences, particularly Alzheimer’s disease research, the general concept of “reserve capacity” has been proposed based on the observations of a discordance between the severity of brain pathology and the cognitive manifestations [19–22]. Yet, this concept may be relevant to any situation when the nervous system sustains injury [20]. The neural mechanisms of reserve capacity are not fully understood. However, differences in the resilience of pre-existing networks may play a role such that an individual with more efficient or higher capacity networks could have a greater capacity in tolerating neuropathologic processes [23]. In the context of handedness, one can postulate that the motor networks responsible for controlling the more dexterous dominant hand are perhaps more efficient with a greater functional capacity being present as a result of use-dependent plasticity compared to the networks associated with controlling the non-dominant hand. Interestingly, it has been recently shown that patients with dominant-side onset Parkinson’s disease had fewer motor deficits compared to those with non-dominant-side onset disease despite similar dopamine reductions on dopamine transporter (DAT) imaging [24]. Likewise, patients with affected dominant hands by cerebrovascular accidents were found to have less motor impairment post-stroke compared to those with the non-dominant hand affected [25]. Our results therefore appear to be consistent with prior observations from neurological disorders. Our findings also have important implications for clinical practice, highlighting the importance of closer examination of the less dexterous non-dominant hand where impairments of fine motor control may be more discernable at an earlier stage of the disease process.

Our findings of greater inter-evaluator reliabilities amongst neurologists compared to neurology residents and APPs is not unexpected, given a greater level of experience in the field and familiarity with the assessment. This emphasizes the importance of enhancing training in neurological exam skills both for neurology residents, as emerging future neurologists, and for the growing number of APPs being integrated within neurological practices to improve the identification of clinical abnormalities [26, 27].

Motor dysfunction in MS, while having a complex substrate which cannot be attributed to a single neuroimaging correlate, has been suggested to be the consequence of infratentorial and spinal cord lesions, as well as damage to the corticospinal tract [13]. In particular, the mean upper cervical cord area, and the presence of cervical and infratentorial lesions can be used relatively easily in the real-world practice setting to predict motor dysfunction [13]. We therefore focused on infratentorial and cervical spinal cord demyelinating lesions in this study.

Our study represents a rigorous attempt to systematically evaluate the value of a classical neurological examination test for detecting infratentorial or cervical spinal cord demyelinating lesions. The other strengths include blinding of evaluators to the study subjects and their clinical and radiological findings, the participation of three categories of healthcare providers in neurology with different levels of training, and experience and a consistent test platform used to acquire our data. Limitations to this effort include a modest number of study subjects that was dictated principally by the anticipated duration of testing (average time for the assessment of 140 videos by each evaluator was 1.5 h), the lack of a formalized rating scale describing finger tapping impairments in MS, utilization of the UPDRS (designed for measuring motor impairments in Parkinson’s disease, not impairments related to CNS demyelinating lesions), and evaluator bias as all healthcare providers were selected from a tertiary academic medical center. One may argue the contribution of lesion laterality in justifying our findings. While we agree that it would have been informative to look into the impact of lesion laterality on finger tapping impairments if we had a larger sample size, we do not believe that lesion laterality can explain our findings given the majority of our patients had multifocal lesions, and given the complexity of the motor networks involved when performing fine motor tasks. It would have also been nice to have an additional control group including MS patients without infratentorial or cervical spinal cord lesions, given the non-specific nature of finger tapping test with respect to the precise lesion location.

Attempts to improve upon classic neurological examination techniques have been previously described. A previous study which questioned the reliability and accuracy of the sign of Babinski was performed, suggesting that the speed of foot tapping may serve as a better predictor of upper motor neuron weakness compared to the plantar reflex [28]. Although this finding provoked criticism [29], it encouraged others to further re-evaluate the reliability of the Babinski sign [30], and some other elements of the neurological exam [31]. Our study was an attempt to address the need for scientific evaluation of neurological exam components by focusing on an important test of fine motor control in upper extremities, and its relationship to neuroimaging findings.

## Conclusions

We found that finger tapping against the distal thumb crease of the non-dominant hand, when evaluated by neurologists, was associated with the highest sensitivity for identifying infratentorial and cervical cord demyelinating lesions. Our findings highlight the importance of precise examinations of the non-dominant side where impaired fine motor control secondary to an upper motor injury might be detectable earlier than the dominant side. These data also encourage several lines of future research. Despite being an easy to perform test in clinical practice, repetitive finger tapping task has a complex nature not only from a neural aspect [32, 33] but also from modeling perspective [34]. Characterization of finger tapping by tapping rate, amplitude or rhythm may not reveal all the information that this valuable test can provide [34, 35]. Future research is therefore warranted to improve upon the characterization of tapping features in relation to neurological diseases.

## Abbreviations

APP: Advanced practice provider
CNS: Central nervous system
DAT: Dopamine transporter
EDSS: Expanded disability status scale
MS: Multiple sclerosis
UPDRS: Unified Parkinson’s disease rating scale
